# A preliminary study of movement intensity during a Go/No-Go task and its association with ADHD outcomes and symptom severity

**DOI:** 10.1186/s13034-016-0135-2

**Published:** 2016-12-12

**Authors:** Fenghua Li, Yi Zheng, Stephanie D. Smith, Frederick Shic, Christina C. Moore, Xixi Zheng, Yanjie Qi, Zhengkui Liu, James F. Leckman

**Affiliations:** 1 Key Lab of Mental Health, Institute of Psychology, Chinese Academy of Sciences, 218 South Block, #16 Lincui Road, Chaoyang District, Beijing, 100101 People’s Republic of China; 2 Beijing Institute for Brain Disorders, Beijing Anding Hospital, Capital Medical University, Beijing, People’s Republic of China; 3 Child Study Center, Yale University School of Medicine, I-265 SHM, 230 South Frontage Road, New Haven, CT 06520-7900 USA; 4 Department of Psychology, University of Southern Mississippi, Hattiesburg, MS USA; 5 Department of Psychology, University of Delaware, Newark, DE USA; 6 Chinese Academy of Medical Sciences, Peking Union Medical College Hospital, Peking Union Medical College, Beijing, People’s Republic of China; 7 University of Chinese Academy of Sciences, Beijing, People’s Republic of China; 8 Center for Child Health, Behavior and Development, Seattle Children’s Research Institute, 2001 8th Ave #400, Seattle, WA 98121 USA

**Keywords:** ADHD, Infrared motion tracking system, Microsoft Kinect, Movement intensity, Frequency bands, Biomarker

## Abstract

**Objective:**

At present, there are no well-validated biomarkers for attention-deficit/hyperactivity disorder (ADHD). The present study used an infrared motion tracking system to monitor and record the movement intensity of children and to determine its diagnostic precision for ADHD and its possible associations with ratings of ADHD symptom severity.

**Methods:**

A Microsoft motion sensing camera recorded the movement of children during a modified Go/No-Go Task. Movement intensity measures extracted from these data included a composite measure of total movement intensity (TMI measure) and a movement intensity distribution (MID measure) measure across 15 frequency bands (FB measures). In phase 1 of the study, 30 children diagnosed with ADHD or at subthreshold for ADHD and 30 matched healthy controls were compared to determine if measures of movement intensity successfully distinguished children with ADHD from healthy control children. In phase 2, associations between measures of movement intensity and clinician-rated ADHD symptom severity (Clinical Global Impression Scale [CGI] and the ADHD-Rating Scale IV [ADHD-RS]) were examined in a subset of children with ADHD (n = 14) from the phase I sample.

**Results:**

Both measures of movement intensity were able to distinguish children with ADHD from healthy controls. However, only the measures linked to the 15 pre-determined 1 Hz frequency bands were significantly correlated with both the CGI scores and ADHD-RS total scores.

**Conclusions:**

Preliminary findings suggest that measures of movement intensity, particularly measures linked to the 10–11 and 12–13 Hz frequency bands, have the potential to become valid biomarkers for ADHD.

**Electronic supplementary material:**

The online version of this article (doi:10.1186/s13034-016-0135-2) contains supplementary material, which is available to authorized users.

## Background

Attention-deficit/hyperactivity disorder (ADHD) is a neurodevelopmental disorder, with an estimated prevalence rate of 5.3% worldwide [1]. In the diagnostic and statistical manual of mental disorders 5th edition (DSM 5), ADHD consists of three distinct presentations: inattentive type, hyperactive-impulsive type, and combined type [2]. Multiple methods have been used to diagnose and assess ADHD and its presentations in children, including clinical interviews, symptom rating scales, behavioral observations, and neuropsychological assessments. However, some of these methods are quite subjective as they rely on parent, teacher, and clinician ratings of ADHD symptom severity. It has been suggested that relying on only one of these traditional assessment procedures and not taking a multi-informant, multi-method approach while assessing children’s functioning across multiple settings, which is currently considered the “gold standard” of diagnostic assessment, may contribute to the over-labeling of children with ADHD, the global rise of ADHD diagnoses in recent years, and the surge in prescribing stimulant medication [3, 4]. However, the sole use of ADHD symptom checklists to make diagnostic decisions is not surprising given the “gold standard” can be both costly and time consuming.

As a result, researchers have become increasingly interested in identifying objective assessment procedures for ADHD that are comparable to the “gold standard” and are more likely to put into practice by clinicians. One approach that has gained traction in recent years is the use of motor tracking systems during neuropsychological tasks of attention and response inhibition. Examples include the use of infrared motion tracking systems that record the vertical and horizontal position of reflectors while children complete a continuous performance task [5–13], or actigraphs/accelerometers (i.e., an acceleration sensor that measures the acceleration of specific body regions) that monitor gross motor activity of children by having them wear sensors on specified locations of their body (e.g., wrist, waist) [14–18]. Martín-Martínez et al. [19] were able to identify children with ADHD combined type by means of a nonlinear analysis of 24-h-long actigraphic registries. Although this method of classification achieved adequate to good precision (Area Under receiver operating characteristic Curve [AUC] values between 0.812 and 0.891), it required an entire 24-h interval of actigraphic data to reach practical diagnostic capabilities. The need for this amount of movement data to make accurate diagnostic predictions is perhaps not surprising, as the actigraph only captures movement as generated by one or two locations on the body rather than simultaneously capturing movements of the entire body. Although currently available actigraph devices can (and do) record temporal or spatial information (e.g., [14], this information has typically been lost in prior studies of children with ADHD due to the way the data were handled and analyzed.

In contrast, infrared motion tracking systems have been previously shown to discriminate boys with ADHD from healthy controls; to correlate with teachers’ ADHD symptom severity ratings and measures of treatment response; and to identify medication doses that produce the best overall clinical results [7, 12, 20, 21]. The data acquired from infrared motion tracking systems are time-locked and able to record the path of movement (i.e., linear versus complex movement patterns); however, methods for integrating movement data across sensors have yet to be developed or reported (instead data from each sensor is reported separately), which potentially limits the precision of these data. In fact, a discrimination analysis of the complexity of head movements did less well in correctly identifying children with ADHD inattentive type from healthy controls (75% of cases correctly classified) than it did with other ADHD presentations. Moreover, head movement data did not significantly correlate with *parent* ratings of ADHD symptom severity [9]. At this time, no known studies have examined the relationship between body movement data as captured by infrared tracking systems and hyperactive/impulsive versus inattentive symptoms. If whole body movements are simultaneously tracked and integrated, such a measure may be sensitive enough to align with severity ratings of inattention since more movement is expected as attention diminishes.

The present study is the first to extract movement intensity measures from recordings of *whole body movements* and to examine whether these measures might be potential biomarkers for ADHD. A biomarker is a directly measurable indicator that may be used to diagnose, evaluate, and monitor the course of a disease as well as predict treatment response [22, 23]. To achieve this goal, movement data tracked and recorded by a Microsoft Kinect System during a Go/No-Go task were analyzed using state-of-the-art signal processing strategies that made use of all available data. It was expected that the Kinect system’s ability to capture and integrate whole body movements would increase the precision with which children with ADHD are identified and be sensitive enough to correlate with symptoms of inattention and hyperactivity/impulsivity.

## Methods

### Study design

This was a two-phase cross-sectional study. The first phase included both an ADHD and a control group to assess the discriminating capabilities of movement intensity measures extracted from data collected by a Microsoft Kinect System. The second phase of the study included only a subset of the ADHD group and was designed to explore associations between movement intensity measures and ADHD symptomatology.

### Participants

Subjects were girls and boys aged 6–12 years living in Beijing city. Children in the ADHD group were selected to participate if they met diagnostic criteria for any presentation of ADHD (inattentive, hyperactive-impulsive, or combined) according to DSM-5 criteria [2] or who were considered to be subthreshold for ADHD, defined as one symptom short of meeting diagnostic criteria. Children with ADHD were excluded if any other co-morbid psychiatric condition (e.g., anxiety disorder, depression) was present. A subset of ADHD cases (N = 14) were recruited from a randomized, wait-list controlled, multi-site study entitled, the “Integrated Brain, Body and Social (IBBS) Intervention for Attention-Deficit/Hyperactivity Disorder” (ClinicalTrials.gov Identifier: NCT01542528; IBBS study) [24] whereas the rest of the ADHD participants (N = 16) were outpatients from a psychiatric hospital serving Beijing City. Children in the control group were matched to children in the ADHD group according to age and gender and were recruited from a local elementary school.

A total of 60 children were enrolled in phase I of the study. Thirty children were in the ADHD group and 30 children were in the control group. All participants were of Han ancestry and each group consisted of 28 boys and 2 girls. The mean age for both groups was 8.95 years (SD = 1.88). The ADHD group consisted of 19 children with ADHD combined type, 5 with inattentive type, 4 with hyperactive-impulsive type, 1 with subthreshold combined type, and 1 with subthreshold inattentive type based on in-person clinical evaluations. One child in the ADHD group had discontinued treatment with methylphenidate (10 mg) due to side effects for 6 months prior to participation in the study.

In phase 2, a total of 14 children from the IBBS study with ADHD or subthreshold for ADHD (9 ADHD combined type, 1 inattentive type, 2 hyperactive-impulsive type, 1 subthreshold combined type, and 1 subthreshold inattentive type) participated. The mean age of the sample was 7.32 years (SD = 1.02). Except for the one child referred to above, all participants were medication naive. Considering ADHD symptom severity ratings were completed only for participants from the IBBS study as part of the assessment protocol and not for those participants recruited from the outpatient clinic, the sample in phase II of the study was limited to just the IBBS study participants.

### Measures

#### ADHD symptom severity

ADHD symptoms were assessed using the ADHD Rating Scale IV (ADHD-RS, [25]). The ADHD-RS has been used repeatedly in the extant literature as a primary outcome measure in ADHD clinical trials (e.g., [26, 27]). Internationally, this scale has been shown to have acceptable psychometric properties [25]. It is comprised of 26 items where 18 items assess ADHD symptoms (9 inattentive, 9 hyperactive/impulsive) and 8 items assess ODD symptoms on a 4-point scale (0 = not at all, 1 = just a little, 2 = quite a bit, 3 = very much). A total composite score is calculated by summing all 18 ADHD items and two subscale scores are derived by separately summing the 9 inattentive and 9 hyperactive/impulsive items. The Clinical Global Impression-Severity (CGI-S) scale also served as a measure of ADHD symptom severity [28]. The CGI-S is rated on a 7-point scale with the severity of illness ranging from 1 (normal) to 7 (amongst the most severely ill patients).

#### Modified Go/No-Go task

This task is a well-known measure of children’s sustained attention and response inhibition ([7, 8, 12, 13, 20, 21, 29–32]. In this version of the Go/No-Go task, a white block appeared inside of a white frame on a black background. A white block appearing at the top of the frame was the “go condition” and a white block appearing at the bottom of the frame was the “no-go condition”. Children were instructed to click the mouse during “go conditions” and to refrain from clicking the mouse during “no-go conditions”. The duration of each stimulus presentation was 500 ms with an inter-trial interval of 1000 ms. Prior to initiating the task, participants were asked if they could see the screen clearly and if their answer was in the affirmative, they were required to complete a minimum of at least five trials with an accuracy of >90% in order for their data to be included. Children were then asked to complete two runs that consisted of 28 blocks (total blocks = 56; 9 trials per block). The first run had a Go/No-Go ratio of 2:7, the second run had a ratio of 7:2. The whole task took approximately 12.6 min to complete (total Go trials = 252, total No-Go trials = 252).

The performance measures of interest for this task included: (i) omission errors (no response given during “Go” trials); (ii) commission errors (response given during “No-Go” trials), (iii) accuracy (correct response across “Go” and “No-Go” trials); (iv) multiple response errors (multiple responses given after stimulus presentation during “Go” trials); (v) reaction time (time it takes to provide a response during “Go” trials); and (vi) reaction time variability (standard deviation of reaction time).

### Measures of movement intensity associated with bodily motion

**Fig. 1:**
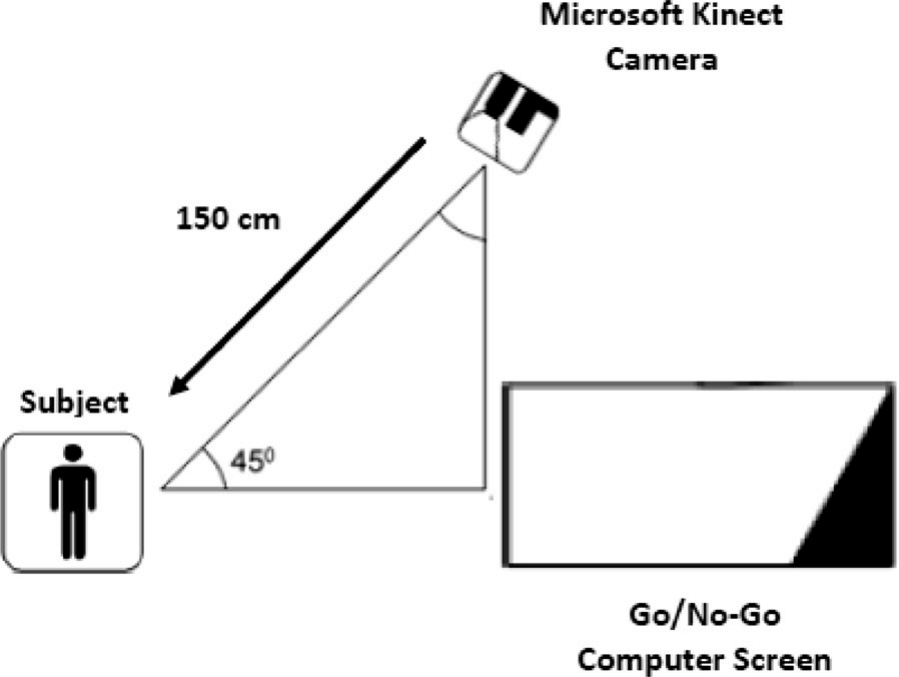
Physical layout for the study

Body movements during a Go/No-Go task were monitored and recorded by a Microsoft Kinect infrared motion sensing camera. This camera was placed 150 cm from the child at a 45^°^ angle from the line between the child and a laptop computer that was used to present the Go/No-Go task (Fig. 1). To ensure the quality of sampling, children were restricted to standing in a circle with a radius of approximately 25 cm [33]. The Kinect camera is a horizontal bar connected to a small base with a motorized pivot and consists of a Red–Green–Blue camera and depth sensor. The camera has a pixel resolution of 640 × 480 and a frame rate of 30 frames per second (FPS). The image depth sensor contains a monochrome complementary metal oxide semiconductor (CMOS) and an infrared projector, which emits multiple infrared rays to form a close-spaced light spot matrix in order to determine its distances from multiple reference points of a participant’s silhouette. The data from this depth sensor were then pre-processed to create a 3-dimensional bitmap that allowed for the monitoring of pixels by comparing temporally adjacent frames to detect movement and extract measures of movement intensity [34].

### Procedures

Both phases of this study were approved by an ethics review board (Scientific Research Ethics Committee of the Institute of Psychology, Chinese Academy of Sciences Beijing, P.R. China). Informed consent was obtained from parents and all child participants gave informed assent prior to initiating any study procedures. For those ADHD participants recruited from the IBBS study, best-estimate DSM-5 diagnoses were assigned by two experienced psychiatrists following a clinical interview with participants’ parents using the Chinese version of the Kiddie Schedule for Affective Disorders and Schizophrenia—Present and Lifetime Version (K-SADS-PL, [35, 36]). ADHD symptom severity ratings were also provided by two expert clinicians as part of the IBBS assessment battery. Once study eligibility was confirmed, participants completed a Go/No-Go Task while the Microsoft Kinect System monitored and recorded their bodily movements. All study procedures for this subset of ADHD participants including the collection of movement data occurred during the IBBS screening visit. The collection of movement data for the remaining ADHD participants took place after their diagnoses were confirmed at the outpatient psychiatric hospital. Diagnoses were made by two experienced psychiatric clinicians based on a clinical interview with the children’s parents, parents’ ratings on a measure assessing their children’s emotions and behavior (i.e., Achenbach Child Behavior Checklist [16]) and an attention task (i.e., Cross-out task [37]). Children from the control group participated in study procedures during one visit to their school by the research team after written consent/assent was given. To confirm the typical development of participating children, their clinical files containing classroom behavior history and routine mental health sessions were reviewed by the school psychologist. A brief screening interview of DSM-5 diagnoses was also done independently by an experienced psychiatrist at the local hospital to confirm their “healthy control” designation. All the movement data were collected in private rooms with the curtains drawn to limit distractions and control the environment’s light so that the children could see the monitor screen clearly.

### Preprocessing of Microsoft Kinect data

This study used bitmap source data of participants’ silhouettes including depth information from the Microsoft Kinect system. The raw silhouette data can be quite unstable and inconsistencies can be observed when viewing the frames in sequence, as noise fragments can be observed bursting across the silhouette even when participants are standing completely still. The noise level of Microsoft Kinect’s infrared sensor has shown to be correlated with the distance between the sensor and target [38] so by keeping this distance constant, one source of noise was minimized. To further account for the remaining noise, a denoise procedure was used to extract the movement intensity measures. First, a baseline assessment of movement was conducted by asking participants to stand still for 15 s. As the average noise level across all 60 participants was 25 pixels (SD = 3.1) when standing still, a scan-line algorithm was used to remove regions of noise smaller than 25 pixels from each participant’s recording. The Kinect data was then preprocessed by comparing two temporally adjacent bitmaps of the silhouette pixel-by-pixel, to determine if there was a change between the two frames (see Additional file 1: Figure S1). Within a given time interval, if a particular pixel had different spatial coordinate values than the previous frame, the program was instructed to mark it as a moved pixel. This yielded a movement intensity value across two adjacent frames where a greater number of moved pixels was indicative of greater intensity in the movement between two frames.

Considering the total pixel count that represented a child’s body was continually changing due to movement, it was necessary to transform the moved pixel count into a converted score by dividing the total moved pixel count by the total mass of the child’s body (i.e., number of pixels representing the child’s silhouette in the current fame).

This converted value of movement intensity was recorded for each frame. As this value was time-locked, it represented a time domain signal to which a Fourier transformation was applied to produce a movement intensity distribution (MID). Since the Kinect camera has a sampling rate of 30 Hz, the frequency domain resolution was expected to be half this sampling rate, resulting in a 0–15 Hz range. The MID data was then subdivided into 15 non-overlapping 1 Hz frequency bands (FB). Thus, the following measures were calculated from the data captured by the Microsoft Kinect System: a composite measure of total movement intensity (TMI) and a movement intensity distribution (MID) across 15 frequency bands (the FB measures).

### Data analytic plan

#### Phase 1

All data analyses were conducted using R programming language version 3.0.3. Independent two-tailed t tests were conducted to compare the ADHD group and control group on their performance on the Go/No-Go task and on each measure of movement intensity. In order to examine the precision with which the Kinect infrared motion tracking camera differentiated children with ADHD from healthy controls, the area under the ROC (Receiver Operating Characteristic) curve (AUC) for the total movement intensity (TMI) and 15 frequency band (FB) measures was calculated. As defined in the research literature, an AUC between 0.7 and 0.9 has adequate precision whereas an AUC above 0.9 has good precision [39]. As prior studies have evaluated Go/No-Go performance measures as potential indicators of ADHD (e.g., [6], ROC-AUC analyses were performed for these measures as well. Finally, bivariate correlations were conducted to examine associations between measures of movement intensity and Go/No-Go task performance.

#### Phase 2

To further examine the usefulness of the movement intensity measures as potential biomarkers for ADHD, bivariate correlations were run between the movement intensity measures and ADHD symptom severity (e.g., ADHD-RS, CGI-S). Correlations between Go/No-Go task performance measures and ADHD symptom severity were also performed. Finally, in an exploratory analysis, we examined if the same FBs that were associated with the ADHD symptom severity measures were also correlated with the inattentive and hyperactive-impulsive subscale scores of the ADHD-RS. To address the multiple comparison problem, the false discovery rate (FDR) method was applied to all *p* values resulting from tests of group differences and correlational analyses.

## Results

### Phase 1

**Fig. 2:**
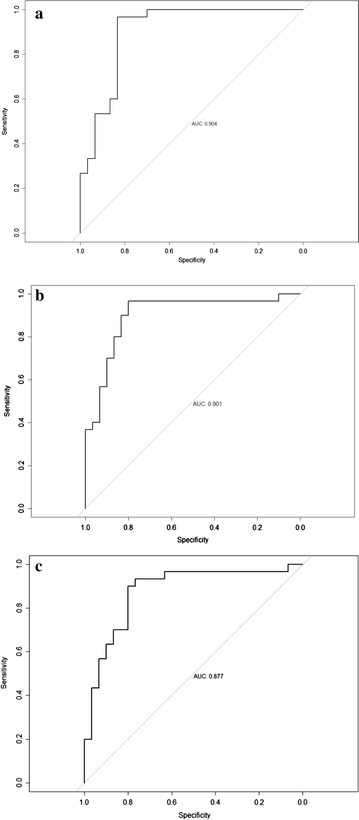
Phase 1: **a** Area under the curve (AUC) of the approximate total movement intensity; **b** AUC of the movement intensity distribution (MID) data for the 10–11 Hz frequency band; and **c** AUC of the MID for the 12–13 Hz frequency band

Children in the control group had significantly better performance across all six performance measures on the Go/No-Go task as compared to the ADHD group (Table 1). The ADHD group displayed more movement than the control group, as group comparisons were all statistically significant (*p* < 0.05) for the TMI and FB measures even after applying the FDR adjustment. The AUC was 0.904 for the TMI measure and between 0.867 and 0.932 for the 15 FB measures indicating that these measures of movement intensity had adequate to good precision with regard to accurately classifying children with and without ADHD (Fig. 2). Overall, 29 of 30 children with ADHD were discriminated from 25 of 30 normal controls with a sensitivity of 0.967 and specificity of 0.833, as calculated using the TMI measure. The ROC-AUC analysis for Go/No-Go task measures revealed AUC values between 0.69 and 0.93 with reaction time variability having the best discriminability: AUC of 0.93, sensitivity of 0.967, and specificity of 0.867. Only commission errors on the Go/No-Go task were significantly correlated with the TMI measure (*r* = 0.28, *p* = 0.03).

**Table 1:** Go/No-Go task performance measures: ADHD group vs. control group

Go/No-Go task measures	ADHD group (n = 30) Mean (SD)	Control group (n = 30) Mean (SD)	t (df)	*p* value	AUC
Omission errors	91.0 (38.9)	63.2 (20.1)	3.47 (43.5)	0.001	0.74
Commission errors	61.4 (16.9)	47.1 (14.2)	3.54 (56.4)	<0.001	0.76
Accuracy	312.6 (63.0)	366.7 (34.9)	−4.12 (45.3)	<0.001	0.844
Multiple responses	39.0 (24.0)	26.9 (8.2)	2.62 (35.7)	0.013	0.69
Reaction time (RT)	580.7 (140.2)	428.1 (37.5)	5.76 (33.1)	<0.001	0.83
RT variability	269.0 (100.1)	100.2 (34.7)	8.73 (35.9)	<0.001	0.929

*SD* standard deviation; *df* degree of freedom; *AUC* area under the curve (an AUC between 0.7 and 0.9 has adequate precision whereas an AUC above 0.9 has good precision)

### Phase 2

After applying the FDR adjustment, 12 out of 15 frequency bands were correlated with the CGI-S scores and 10 out of 15 bands were correlated with the ADHD-RS total scores, 10 out of 15 bands were correlated with the ADHD-RS hyperactivity subscale and 7 out of 15 bands were correlated with the ADHD-RS inattentive subscale. The 10–11 and 12–13 Hz frequency bands had the strongest correlations with the ADHD-RS (total and subscales) and CGI-S scores (Table 2). The TMI measure was not correlated with the ADHD-RS total scores or either the hyperactivity or inattentive subscale scores, but it was significantly correlated with the CGI-S scores (*r* = 0.61, *p* = 0.021). There were no significant correlations between any of the Go/No-Go performance measures and ADHD symptom severity measures [ADHD-RS (total and subscales) and CGI-S].

**Table 2:** Correlations of clinician ratings of ADHD symptom severity (N = 14) and the most promising frequency bands of the movement intensity distributions (MID) measured using the Microsoft Kinetic system

FBs of MID	CGI-S	ADHD-RS Total	ADHD-RS Inattentive	ADHD-RS Hyperactive/impulsive
10–11 Hz	*r* = 0.60 (*p* = 0.006)	*r* = 0.67 (*p* = 0.008)	*r* = 0.63 (*p* = 0.015)	*r* = 0.64 (*p* = 0.014)
12–13 Hz	*r* = 0.65 (*p* = 0.013)	*r* = 0.69 (*p* = 0.006)	*r* = 0.65 (*p* = 0.012)	*r* = 0.65 (*p* = 0.011)
Total movement intensity	*r* = 0.61 (*p* = 0.002)	*r* = 0.53 (*p* = 0.051)	*r* = 0.50 (*p* = 0.067)	*r* = 0.50 (*p* = 0.069)

*FBs* frequency bands; *MID* movement intensity distributions; *CGI-S* the clinical global impression severity; *ADHD-RS* ADHD rating scale

## Discussion

The purpose of this study was to use an infrared motion tracking system to monitor and record the movement intensity of children in order to determine its diagnostic precision for ADHD and its possible association with ratings of ADHD symptom severity. Results from this study revealed that our measures of movement intensity [i.e., a composite measure of total movement intensity (TMI measure) and a movement intensity distribution measure across 15 frequency bands (FB measures)] were able to distinguish children with ADHD from healthy controls. However, only the measures linked to the 15 pre-determined 1 Hz frequency bands were significantly correlated with both the CGI scores and ADHD-RS total scores. The 10–11 and 12–13 Hz frequency bands had the strongest correlations with these ADHD symptom severity measures. Both of these frequency bands were also significantly associated with the inattentive and hyperactive/impulsive subscales of the ADHD-RS. The following discussion considers potential implications for these findings, limitations of this study’s design, and future research directions.

The first phase of this study examined the discriminating capabilities of our movement intensity measures with respect to children with ADHD and healthy control children. Our results aligned well with prior studies using other measures extracted from movement data, as children with ADHD performed less well and engaged in more movement than healthy control children when completing a neuropsychological task of attention and response inhibition while their body movements were recorded [20, 29]. In contrast to our predictions, our movement intensity measures did not outperform, but instead, were comparable in terms of their ability to differentiate children with ADHD from healthy controls [6, 12, 19]. Interestingly, the only Go/No-Go performance measure to match the discriminating capabilities of our movement intensity measures was reaction time variability, which has been identified as a stable feature of ADHD in a recent meta-analytic review [40]. However, the only Go/No-Go performance measure to significantly correlate with our measures of movement intensity was commission errors, which suggests that our findings (e.g., correlations between movement intensity measures and ADHD symptom severity ratings) are not attributable to the Go/No-Go task and these performance and movement intensity measures are potentially tapping different aspects of ADHD.

It is also worth noting that the measure of movement intensity used in this study achieved a better classification accuracy than did a functional neuroimaging procedure using functional near-infrared spectroscopy (fNIRS) during the course of a Go/No-Go task [41]. This suggests that the movement intensity procedures used in this study *might* be an effective biomarker for children with ADHD at the individual level. More specifically, we are interested in determining whether measures of movement may contribute to a clinician’s ability to diagnose, evaluate, and monitor a disease, as well as track an individual’s response to treatment [22, 23].

The second phase of this study was aimed to further examine the usefulness of measures of movement intensity as potential biomarkers for ADHD by looking at associations between these movement intensity measures and ADHD symptom severity. As predicted, our measures of movement intensity were significantly correlated with overall ADHD symptom severity in addition to symptoms of hyperactivity/impulsivity and inattention whereas movement measures isolated to one location of the body are not [12]. Indeed, a more stringent test to evaluate the potential of our movement intensity measures as ADHD biomarkers was employed since *clinician*-*rated* measures of ADHD symptom severity were used, which are considered more objective than parent or teacher ratings. In contrast, the Go/No-Go performance measures failed to significantly correlate with any measures of ADHD symptom severity. These findings underscore the potential value of monitoring movement intensity associated with body movements, over and above neuropsychological tasks of attention and response inhibition, to objectively assess ADHD symptom severity over time and in response to treatment. However, our results need to be replicated by comparing the discriminating capabilities of the movement intensity measures to other neuropsychological tasks (e.g., continuous performance task) before any firm conclusions can be made.

Another novel approach used in this study concerns the potential value of movement intensity measures that are linked to specific frequency bands. Our preliminary data indicate that the 10–11 and 12–13 Hz frequency bands are particularly promising. One possible explanation for the strong correlations found between these specific frequency bands and clinician ratings of ADHD symptoms is that the high frequency signals, after Fourier transformation, reflect minor waves of movement intensity that are associated with small movements like fidgeting actions of the fingers or partial body discordant movements. Such a possibility highlights the sensitivity of this particular measure and its potential clinical utility.

Another finding that deserves some attention is that the total movement intensity measure did not correlate with most measures of ADHD symptom severity. A possible explanation for this finding could be that the body movements associated with ADHD were only reflected in a portion of the frequency bands and the total movement intensity is the sum of all frequency bands. This also provides preliminary support that a frequency domain perspective may be a more refined approach to monitor ADHD-related body movements.

## Future directions and limitations

ADHD is frequently comorbid with other neurodevelopmental and neuropsychiatric disorders including oppositional defiant disorder, conduct disorder, Tourette syndrome, depression, anxiety disorder, and learning disorders [42]. Future studies are needed to determine the degree to which these co-occurring disorders have an impact on estimates of movement intensity. This may be particularly problematic for movement disorders like Tourette’s Disorder which is highly comorbid with ADHD [43]. Given Tourette’s Disorder is a movement disorder, it would be difficult to partition out which movements are attributable to Tourette’s and which are attributable to ADHD using the current methods described in this study. However, applying more morphologic and pattern recognition methods to movement data of children with ADHD and Tourette’s may potentially enable us to identify their distinct attributes or even build computer vision classifiers. Relatedly, it would be worthwhile to use infrared motion tracking technology to identify movement patterns of other mental disorders in order to isolate those patterns that are specific to ADHD. Similar approaches are underway with fNIRS as well as volumetric and functional MRI data from individuals with a range of neuropsychiatric disorders including ADHD [41, 44, 45].

In this study, we recruited participants with ADHD across all diagnostic presentations. However, we did not compare differences in movement intensity across presentations because of our limited sample size. Future research should consider determining whether or not our movement intensity measures are capable of differentiating children across ADHD presentations. Longitudinal studies are also needed to examine the test–retest reliability of these measurements as well as their ability to monitor symptom severity over time. Indeed, a key question concerns the sensitivity of this measure to detect clinical improvement following treatment. Assessing simultaneously measures of movement intensity and fNIRS in regions identified in the right prefrontal cortex during a Go/No-Go task, as was done in a previous study, might be another promising line of research [41].

With respect to study limitations, we compared our movement intensity measures to a multi-method clinician-driven method of diagnostic classification, which is an approach commonly used in clinical trials [7, 18, 46]; however, it is important to point out that this is not considered the “gold standard” of ADHD assessment. Therefore, future studies should consider comparing these measures of movement intensity to this “gold standard” (e.g., multi-informant, multi-method evaluation of functioning across multiple settings) to further evaluate its diagnostic precision. It should also be noted that our Go/No-Go task had an equivalent number of Go and No-Go trials across the entirety of the paradigm; however, the Go trials were five times more frequent than the No-Go trials in the second run of the task, thus capturing response inhibition. In future studies, it is recommended that the number of Go trials always exceed the number of No-Go trials in order to optimize response inhibition. Finally, as with all methods of assessment, our measures of movement intensity are not without error. Data quality was limited due to the noise of the image signal and a sparse light structure sampling coverage with a frame rate of 30 Hz, thus limiting granularity of the data. Also, the frame-to-frame comparison algorithm may have underestimated movement for the x–y coordinate axes and overestimated for the z-coordinate axis. By using a more precise data collection device (e.g. laser scanner) and surface and voxel-based rebuild tracking techniques, there may be considerable precision improvement. It may also be useful to simultaneously record body movements of participants with a visible light band camera to further assess the nature of their movements via qualitative analysis software.

## Conclusion

Locomotor activity and movement intensity are emerging as core constructs in our understanding of ADHD. In this study, movement intensity measures extracted from body movement data by an infrared motion-sensing camera during a Go/No-Go task was found to distinguish children with ADHD from typically developing children and to be highly correlated with clinician ratings of symptom severity. These results suggest that using infrared motion detecting systems to calculate measures of movement intensity has the potential to become a useful clinical tool that may have several advantages over traditional approaches. Specifically, these methods have the potential to be more time and cost efficient than the “gold standard” of ADHD assessment, thus enhancing the likelihood of clinicians making use of this objective indicator without relying on single informant measures that are subject to biases. These advantages highlight the importance of replication studies, as movement intensity measures extracted from body movements may prove to be a new behavioral biomarker of ADHD.

## Additional files

**Additional file 1: Figure S1.** A Sequence diagram of the program used to analyze the Microsoft Kinect Data. This is the sequence diagram of the computer program used to analyze the Microsoft Kinect data. The component processes are connected by the arrows from left to right. The vertical direction shows the lifecycle for the timeline for each process.

## Abbreviations

ADHD: attention-deficit/hyperactivity disorder
TMI: total movement intensity
FB: frequency bands
CGI: clinical global impression scale
ADHD-RS: ADHD-rating scale
DSM 5: diagnostic and statistical manual of mental disorders 5th edition
AUC: area under receiver operating characteristic curve
IBBS: integrated brain, body and social intervention
CGI-S: clinical global impression-severity
FPS: frames per second
COMS: complementary metal oxide semiconductor
K-SADS-PL: Kiddie schedule for affective disorders and schizophrenia-present and lifetime version
MID: movement intensity distribution
ROC: receiver operating characteristic
FDR: false discovery rate
fNIRS: functional near-infrared spectroscopy
