# Supplementary material for: A preliminary study of movement intensity during a Go/No-Go task and its association with ADHD outcomes and symptom severity
Source: Child Adolesc Psychiatry Ment Health. 2016 Dec 12;10:47. doi: 10.1186/s13034-016-0135-2 (PMC5153899; doi:10.1186/s13034-016-0135-2)
Supplement: Supplementary file 1 [file 13034_2016_135_MOESM1_ESM.docx]

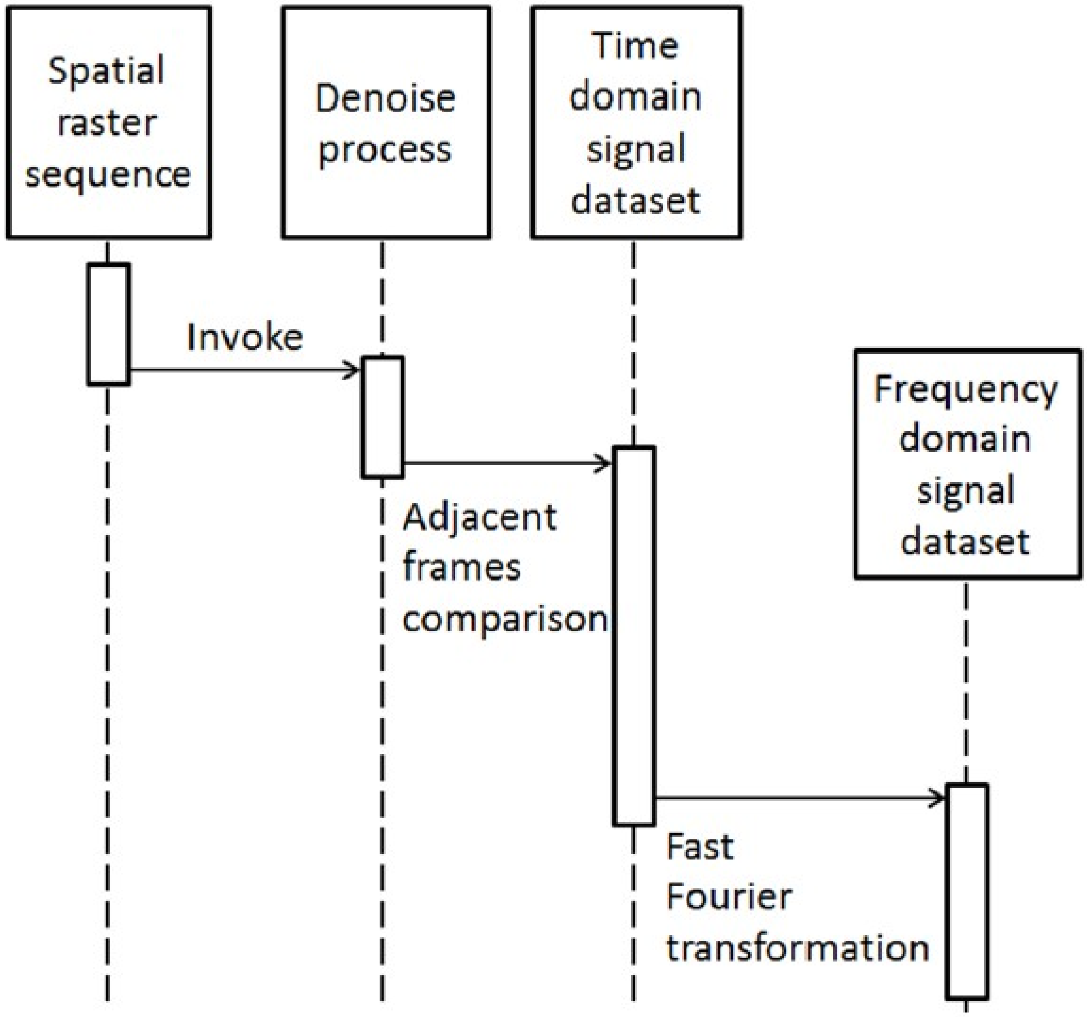

Spatial raster sequence
Denoise process
Time domain signal dataset
Frequency domain signal dataset
Invoke
Adjacent frames comparison
Fast Fourier transformation
